# Cat (Fel d 1) and dog (Can f 1) allergen levels in cars, dwellings and schools

**DOI:** 10.1007/s10453-016-9433-7

**Published:** 2016-03-11

**Authors:** A. Niesler, G. Ścigała, B. Łudzeń-Izbińska

**Affiliations:** Department of Biohazards and Immunoallergology, Institute of Occupational Medicine and Environmental Health, 13 Kościelna Street, 41-200 Sosnowiec, Poland

**Keywords:** Cat and dog allergens, Environmental exposure, Car, School and home environments, Dust, Airborne, Pet ownership

## Abstract

Pets are an important source of indoor allergens. The aim of the study was to compare cat and dog allergen levels in cars, schools and homes. The study was carried out in 17 cars, 14 classrooms and 19 dwellings located in the highly industrialized and urbanized region of Poland. Dust and air samples were analyzed for Fel d 1 and Can f 1 using a double monoclonal ELISA assay. The highest amounts of cat and dog allergens (Fel d 1: 1169 μg/g; Can f 1: 277 μg/g) were found in dwellings with pets. Allergen concentrations were correlated with the number of animals kept at home. Although concentrations on automobile seats were lower, Fel d 1 levels exceeded 8 μg/g in 23.5 % of cars and high levels of Can f 1 (>10 μg/g) were found in 17.6 % of cars. The study revealed that cars of pet owners may be reservoirs of cat and dog allergens even when animals are not transported in them. In schools, concentrations of pet allergens did not reach high levels, but the moderate levels of Fel d 1 (≥1–8 μg/g) and Can f 1 (≥2–10 μg/g) were detected in 42.9 and 7.1 % of the investigated classrooms. Concentrations of cat and dog allergen in schools were higher than in homes without pets. While airborne Fel d 1 and Can f 1 levels were found low, residential allergen concentrations in settled dust and air were correlated. The study results suggest that classrooms and cars of pet owners may be important sites of exposure to cat and dog allergens, though the highest concentrations of Fel d 1 and Can f 1 are found in homes of pet owners.

## Background

Over the last few decades, the prevalence of asthma and allergic diseases has increased (Johnson et al. 2002; Maziak et al. 2003; de Marco et al. 2012; Qu et al. 2013). Furthermore, a large geographical variation in symptoms has been observed (Janson et al. 2001; Asher et al. 2006). In the Polish population, asthma and allergic disorders are serious problems of public health (Liebhart et al. 2007; Samoliński et al. 2009; Brożek et al. 2010). In a number of studies, an increase in the prevalence of allergic diseases in urban areas compared to rural ones has been observed (Nicolaou et al. 2005; Majkowska-Wojciechowska et al. 2007). The role of environmental factors in development of respiratory symptoms is significant (Kasznia-Kocot et al. 2010; Heinrich 2011). Several studies have confirmed that exposure to animal allergens is an important risk factor for eczema and allergic respiratory diseases (Brussee et al. 2005; Brunekreef et al. 2012). Greater asthma severity in allergic individuals is associated with increased pets allergen concentrations (Salo et al. 2008; Gent et al. 2009). However, direct relationship between the level of exposure to allergens, prevalence of sensitization to pets and development of asthma remains problematic (Custovic et al. 2010; Platts-Mills and Woodfolk 2011). Moreover, exposure to animals can develop a form of tolerance without causing allergic disease (Murray et al. 2001).

According to the Public Opinion Research Center (Wciórka 2003), 52 % of polish households include at least one pet. The most favored are cats (19 %) and dogs (36 %). The literature data focused on environmental exposure to pet allergens are very limited, in Poland. Up to the moment, studies were carried out only in homes (Jedrychowski et al. 2008; Waradzyńska et al. 2012; Kozajda et al. 2013) and kindergartens (Cyprowski et al. 2013). Depending on the presence of pets, three different environments were chosen for our study. Permanent presence of the animal (households with cat or dog), temporary staying (transporting pets inside the car) and no animals residing (classrooms, households without pet ownership) were taken into consideration. Thus, the aim of the study was to compare cat and dog allergen levels in cars, schools and homes.

## Materials and methods

The study was carried out in 17 cars, 14 classrooms of 6 schools and 19 dwellings located in the Upper Silesia—the highly industrialized and urbanized region of southern Poland. Variations in the location, age and technical condition of the building as well as number of children were the main criteria for choosing a school. Two of the schools were situated outside the agglomeration, 2 schools in the suburbs and 2 schools in the city center. The mean age of the buildings was 49 years (range 20–85 years). The number of children attending the surveyed schools varied from 60 to 391, and the number of pupils per classroom ranged from 9 to 114 (in classroom with rotation system). The number of cat and dog owners in investigated classrooms varied from 0 to 14 and from 1 to 39, respectively. Pet ownership and transporting animal inside the car were the most important criteria for choosing a home. Of the investigated homes, 11 contained cats and 10 dogs. One of the households did not own pets, and in three of the surveyed homes, both a cat and a dog were present. Six cat owners and six dog owners used their car for animal transport. Selected homes, as well as schools, differed in location (1 home outside the agglomeration, 12 in the suburbs and 6 in the city center) and age of the building (range 6–100 years). Of the selected homes, there were 12 detached houses and 7 flats in an apartment block.

### Settled dust and air sampling

Dust samples were collected with 2100 W vacuum cleaner (Zelmer S.A. Rzeszów, Poland) using a specially constructed dust trap filter. A surface area of 2 m^2^ of smooth and carpeted floor was vacuumed for 2 min, in classrooms and dwellings (usually in living room and bedroom). In dwellings, samples were also taken from beds, upholstered furniture and dens of pets. In cars, whole surface of seats were vacuumed for 2 min. A total number of 133 dust samples were collected (including 17 samples from cars, 22 samples from classrooms and 94 from dwellings) from which 115 were examined for cat allergen (Fel d 1) and 126 for dog allergen (Can f 1).

Air samples were taken at the height of 1.0–1.5 m above floor level to simulate aspiration from the human breathing zone, using a Casella Vortex ultraflow pumps (Casella, Amherst, USA) on polyvinylidene fluoride membrane filters (0.45 μm, 25 mm diameter; Millipore Corp., Billerica, MA). Samples were taken at flow rates of 4 and 8 L/min. The volume of the air sampled was 3–18 m^3^. A total number of 21 air samples were collected and examined, including 6 samples from classrooms (from 6 schools) and 15 from dwellings (usually from living room or bedroom). In 4 homes, we had no permission to perform measurements (due to long sampling time and generated noise).

### Processing of samples and allergen measurements

All samples were prepared for the ELISA assay in accordance with test manufacturer instructions (Indoor Biotechnologies Ltd, Warminster, UK). Dust samples were sieved through a 355-μm-diameter mesh screen to remove large particles and fibers. After weighing 100 mg of “fine dust” obtained, settled dust samples and filters were extracted by shaking for 2 h with suitable amount of PBS-T (phosphate-buffered saline with 0.05 % Tween 20, pH 7.4). Extracts were centrifuged for 20 min at 2500 rpm. Supernatants were stored at −20 °C until analyzed.

The concentration of cat (Fel d 1) and dog (Can f 1) allergens was evaluated using a double monoclonal ELISA assay. It was performed according to the protocol from the manufacturer (Indoor Biotechnologies Ltd, Warminster, UK), using BIO-TEK microplate reader (ELx808TM Microplate Reader, BIO-TEK Instruments, INC., Vermont, USA). Dust extracts were initially assayed at 5-, 25- and 125-fold dilution for cars, schools and homes without pets, and at 100-, 500- and 2500-fold dilution for homes with pets. Air samples were assayed neat and two-, four- and eightfold dilution. Each sample was measured in duplicate. Because some samples had too little dust to analyze both allergens, there were missing Fel d 1 and Can f 1 values (in such cases, in homes with cat only Fel d 1 was measured, samples from homes with dog were assayed for Can f 1 only). Concentrations were expressed as microgram of allergen (Fel d 1 or Can f 1) per gram of dust (dust samples) and nanogram of allergen per cubic meter of air (air samples).

Moderate and high levels of allergens were determined based on levels identified in the literature as potentially related to sensitization or asthma exacerbation (Ingram et al. 1995; Leaderer et al. 2002). Lower and upper cut points of the distribution for allergens were ≥1 and ≥8 μg/g for Fel d 1 and ≥2 and ≥10 μg/g for Can f 1 (Tables 1, 2).

**Table 1:** Concentration of cat allergen (Fel d 1) in settled dust samples

Sampling locations		Number of samples	Number of samples < LLOD (%)	Fel d 1 concentration [μg/g]		Level of Fel d 1 allergen [Number of samples (%)]	
				GM	Range	Moderate level ≥ 1–8 μg/g	High level ≥ 8 μg/g
Cars	Seats	6^t^	–	4.46^t^	0.76–14.17^t^	3 (50)^t^	2 (33.3)^t^
		4^nt^	–	3.33^nt^	1.0–8.74^nt^	1 (25)^nt^	2 (50)^nt^
		7^#^	1 (14.3)^#^	0.23^#^	0.02–1.07^#^	2 (28.6)^#^	–
	Total	17	1 (5.9)	0.97	0.02–14.17	6 (35.3)	4 (23.5)
Schools	Smooth floor	14	–	0.25	0.06–3.3	3 (21.4)	–
	Carpeted floor	8	–	0.68	0.12–3.13	4 (50)	–
	Total	22	–	0.36	0.06–3.3	7 (31.8)	–
Dwellings	Beds	9*	–	15.01*	0.05–499.13*	3 (33.3)*	5 (55.6) *
		2**	–	1.49**	0.59–3.74**	1 (50)**	–
		6^#^	1 (16.7)^#^	0.04^#^	0.02–0.47^#^	–	–
	Upholstered furniture	8*	–	51.8*	0.25–406.16*	–	7 (87.5)*
		2**	–	3.18**	1.89–5.33**	2 (100)**	–
		6^#^	–	0.15^#^	0.02–0.48^#^	–	–
	Dens of pets	7*	–	144.19*	3.92–1169.16*	1 (14.3)*	6 (85.7)*
	Smooth floor	6*	–	17.89*	0.13–484.73*	1 (16.7)*	4 (66.7)*
		1**	–	0.34**^a^	NA	–	–
		5^#^	–	0.07^#^	0.04–0.13^#^	–	–
	Carpeted floor	11*	–	11.07*	0.03–520.83*	3 (27.3)*	7 (63.6)*
		4**	–	1.88**	0.62–10.14**	1 (25)**	1 (25)**
		9^#^	2 (22.2)^#^	0.05^#^	0.02–0.78^#^	–	–
	Total	76	3 (3.9)	2.46	0.02–1169.16	12 (15.8)	30 (39.5)

<*LLOD* Below the lower limit of detection, *GM* geometric mean, *a* single measurement, *NA* not available, *t* cat transported inside the car, *nt* cat owner does not transport it by car

* Homes with cat inside, ** homes with cat outside only, ^#^ no cat at home

**Table 2:** Concentration of dog allergen (Can f 1) in settled dust samples

Sampling locations		Number of samples	Number of samples < LLOD (%)	Can f 1 concentration [μg/g]		Level of Can f 1 allergen [Number of samples (%)]	
				GM	Range	Moderate level ≥ 2–10 μg/g	High level ≥ 10 μg/g
Cars	Seats	6^t^	–	3.87^t^	0.63–14.32^t^	2 (33.3)^t^	2 (33.3)^t^
		2^nt^	–	1.24^nt^	0.12–13.134^nt^	–	1 (50)^nt^
		9^#^	1 (11.1)^#^	0.37^#^	0.11–1.53	–	–
	Total	17	1 (5.9)	0.86	0.11–14.32	2 (11.8)	3 (17.6)
Schools	Smooth floor	14	–	0.46	0.12–1.11	–	–
	Carpeted floor	8	–	0.82	0.25–4.45	1 (12.5)	–
	Total	22	–	0.57	0.12–4.45	1 (4.5)	–
Dwellings	Beds	8*	–	16.4*	2.82–152.71*	3 (37.5)*	5 (62.5)*
		2**	1 (50)**	0.14**	NA	1 (50)**	–
		9^#^	4 (44.4)^#^	0.04^#^	0.1–0.66^#^	–	–
	Upholstered furniture	8*	–	23.57*	1.5–60.70*	1 (12.5)*	6 (75)*
		2**	–	3.07**	0.5–18.77 **	–	1 (50)**
		8^#^	1 (12.5)^#^	0.14^#^	0.01–3.50^#^	1 (12.5)^#^	–
	Dens of pets	8*	–	59.88*	1.26–276.7*	–	7 (87.5)*
	Smooth floor	9*	–	23.96*	10.41 – 138.49*	–	9 (100)*
		1**	–	0.5**^a^	NA	–	–
		5^#^	4 (80)^#^	0.02^#^	NA	–	–
	Carpeted floor	11*	–	33.42*	1.01–204.56*	1 (9.1)*	9 (81.8)*
		4**	–	0.15**	0.03–1.01**	–	–
		12^#^	4 (33.3)^#^	0.06^#^	0.06–0.73^#^	–	–
	Total	87	14 (16.1)	1.56	0.01–276.7	7 (8)	37 (42.5)

<*LLOD* Below the lower limit of detection, *GM* geometric mean, *a* single measurement, *NA* not available, *t* cat transported inside the car, *nt* cat owner does not transport it by car

* Homes with cat inside, ** homes with cat outside only, ^#^ no cat at home

### Statistical analyses

The collected data were statistically elaborated with Shapiro–Wilk, Kruskal–Wallis, Mann–Whitney tests and Spearman rank correlation using Statistica (data analysis software system), version 7.1 (StatSoft, Inc., Tulsa, OK, USA). For statistical analysis, samples with Fel d 1 and Can f 1 allergen levels below the detection were assigned a value of half the limit of detection (which were 0.2 and 0.5 ng/ml, respectively).

## Results

### Settled dust samples

#### Concentration of cat allergen (Fel d 1)

The concentrations of Fel d 1 (detectable in 96.5 % of samples) are presented in Table 1. In cars, concentrations of Fel d 1 ranged from 0.02 to 14.17 μg/g with GM of 0.97 μg/g. Cat allergen levels above the upper cut point (≥8 μg/g) were detected in four cars. The concentrations of Fel d 1 in cars of cat owners were considerably higher than in cars whose owners did not have a cat, regardless of whether the animal was transported inside (Mann–Whitney test: *p* < 0.01 for cars with cat transport declared and *p* < 0.05 for cars without cat transport but cat at home reported). In general, Fel d 1 concentrations in schools (GM 0.36 μg/g, range 0.06–3.3 μg/g) were lower than in cars and dwellings (excluding dens of pets, GM 1.64, range 0.02–520.83 μg/g). The GM per school varied from 0.1 to 2.3 μg/g. Fel d 1 in levels above the lower cut point (≥1 μg/g) were found in 7 samples taken in classrooms. Detectable levels of Fel d 1 were found in 88.5 % of samples taken in homes without cat. In households without pet ownership, concentrations of Fel 1 (GM 0.06 μg/g, range 0.02–0.78 μg/g) were considerably lower than in classrooms and homes with cat (excluding dens of pets, GM 18.78 μg/g, range 0.03–520.83 μg/g) (Mann–Whitney test *p* < 0.01). The GM per home with and without cat varied from 0.08 to 280.51 μg/g (excluding dens of pets) and from <0.01 to 0.48 μg/g, respectively. In 30 samples taken in dwellings, cat allergen concentration was greater than 8 μg/g. There was a significant correlation between the number of cats kept at home and concentration of Fel d 1 (Spearman’s correlation *p* < 0.01, *r* = 0.78). No significant differences in amounts of Fel d 1 on living room and bedroom floors were noted (Mann–Whitney test *p* > 0.05). A comparison of the results obtained from different surfaces in dwellings indicated that the highest Fel d 1 concentrations were found in dust from dens of cats (GM 144.19 μg/g, range 3.92–1169.16 μg/g). They were considerably higher than in samples from floors and beds (Kruskal–Wallis test *p* < 0.05). In homes with cat, the GM level of Fel d 1 exceeded 50 μg/g in dust from upholstered furniture and was greater than 10 μg/g in samples from beds and floors. There were no significant differences in concentration of Fel d 1 in dust from smooth and carpeted floors, both in dwellings and schools (Mann–Whitney test *p* > 0.05).

#### Concentration of dog allergen (Can f 1)

Can f 1 was detected in 88.1 % of samples. The concentrations of Can f 1 are shown in Table 2. In general, concentrations of Can f 1 in schools (GM 0.57 μg/g, range 0.12–4.45 μg/g) were lower than in dwellings (excluding dens of pets, GM 1.08 μg/g, range 0.01–204.56 μg/g) and cars (GM 0.86 μg/g, range 0.11–14.32 μg/g). Dog allergens in levels above the upper cut point (≥10 μg/g) were detected in three cars. The concentrations of Can f 1 in cars whose owners transport dog inside were considerably higher than in cars whose owners did not have a pet (Mann–Whitney test *p* < 0.01). There were no significant differences in amounts of Can f 1 in cars of dog owners depending on whether the animal was transported inside. The GM per school varied from 0.3 to 1.18 μg/g. In one classroom, the level of Can f 1 exceeded 2 μg/g. Detectable levels of Can f 1 were found in 91 % of samples taken in homes without pet, but with reported dog ownership a few years (2–5) before the study and in 48 % of samples taken in homes with negative interview on dog currently and in the past. In dwellings without dog ownership, concentrations of Can f 1 (GM 0.05 μg/g, range 0.01–3.5 μg/g) were considerably lower than in classrooms and homes with dog (excluding dens of pets, GM 24.29 μg/g, range 1.01–204.56 μg/g) (Mann–Whitney test *p* < 0.01). The GM per home with and without dog varied from 2.22 to 84.44 μg/g (excluding dens of pets) and from <0.01 to 0.97 μg/g, respectively. In 37 samples taken in dwellings, Can f 1 concentration was greater than 10 μg/g. The concentrations of Can f 1 were correlated with the number of dogs kept at home (Spearman correlation *p* < 0.01, *r* = 0.83). There were no significant differences in concentration of Can f 1 in dust from living room and bedroom floors (Mann–Whitney test *p* > 0.05). The highest concentrations of Can f 1 were observed in dust from dens of dogs (GM 59.88 μg/g, range 1.26–276.7 μg/g). However, only concentrations obtained from dens, carpeted floors and beds were significantly different (Kruskal–Wallis test *p* < 0.05). Excluding dens of pets, the highest GM level of Can f 1 (>30 μg/g) was found in dust from carpets, in dwellings with dog. In households with pet ownership, the GM level of Can f 1 exceeded 10 μg/g in dust from beds and was greater than 20 μg/g in samples from upholstered furniture and smooth floors. The concentrations of Can f 1 in dust from smooth and carpeted floors in dwellings and schools were not significantly different (Mann–Whitney test *p* > 0.05).

### Air samples

The levels of Fel d 1 and Can f 1 found in air samples are presented in Table 3. Both allergens were detectable in 43 % of samples. Obtained concentrations did not exceed 3 ng/m^3^. Furthermore, none of the air samples taken in schools had detectable level of Fel d 1. Concentrations of airborne Can f 1 found in classrooms and dwellings were not significantly different (Mann–Whitney test *p* > 0.05). There was a significant association between Fel d 1 and Can f 1 levels obtained from dust and air samples in dwellings (Spearman’s correlation *p* < 0.01, *r* = 0.65 and *r* = 0.59, respectively).

**Table 3:** Concentration of cat (Fel d 1) and dog (Can f 1) allergens in air samples

Allergen	Sampling locations	Number of samples	Number of samples < LLOD (%)	Allergen concentration [ng/m^3^]	
				GM	Range
Fel d 1	Schools	6	6 (100)	–	–
	Dwellings	7*	1 (14.3)*	0.35*	0.09–2.21*
		2**	–	0.09**	0.07–0.1**
		6^#^	5 (83.3)^#^	0.04^#^	NA
Can f 1	Schools	6	2 (33.3)	0.3	0.3–0.91
	Dwellings	6*	2 (33.3)*	0.45*	0.72–1.11*
		2**	2 (100)**	0.09**	NA
		7^#^	6 (85.7)^#^	0.06^#^	NA

<*LLOD* Below the lower limit of detection, *GM* geometric mean, *NA* not available

* Homes with cat/dog inside, ** homes with cat/dog outside only, ^#^ no cat/dog at home

## Discussion

The results of the presented study showed that pet allergen levels in cars, schools and dwellings were different. Concentrations of Fel d 1 and Can f 1 in cars were lower than in homes with pets, but higher than in classrooms. Obtained results confirm the role of automobiles in the dispersal of pet allergens (Neal et al. 2002). We found that 23.5 % of cars had Fel d 1 allergen level greater than 8 μg/g. The high level of Can f 1 allergen (≥10 μg/g) was detected in 17.6 % of cars. According to the literature data, such concentrations are considered as risk levels to atopic or sensitized individuals for acute attacks of asthma (Tranter 2005). The levels of cat allergen ≥1–8 μg/g and dog allergen ≥2–10 μg/g are regarded as risk factors for allergic sensitization of genetically predisposed people (Kozajda et al. 2013). In our study moderate levels of Fel d 1 and Can f 1 allergens were found in 35.3 and 11.8 % of cars, respectively. However, dog allergen concentrations were lower than those obtained by Taketomi et al. in private cars (Taketomi et al. 2006). Since cat and dog allergens can be transported on hair and clothes of pet owners (Berge et al. 1998; Almqvist et al. 1999; Karlsson and Renström 2005; Cyprowski et al. 2013), they are frequently detected in environments in which no animals reside (Custovic et al. 1996; Brunetto et al. 2009; Salo et al. 2009). As in case of studies carried out in Brazilian cars (Justino et al. 2005), we have found that concentrations of cat and dog allergens in cars whose owners had a pet were considerably higher than in cars whose owners did not have. However, there were no significant differences in allergen levels regarding whether the owners transported pet inside. High levels of pet allergens were found in both types of car.

Cat and dog allergens have frequently been detected in school environment (Tranter 2005; Salo et al. 2009; Fsadni and Montefort 2013; Zahradnik and Raulf 2014). Moreover, differences in pet allergen concentrations between classes with few and many cat owners were found (Karlsson et al. 2004; Instanes et al. 2005). In our study, all dust samples taken from floors in schools had detectable levels of Fel d 1 and Can f 1, but allergen concentrations were not correlated with the number of pet owners. Moderate levels of Fel d 1 and Can f 1 were detected in 42.9 and 7.1 % of the investigated classrooms, respectively. The study revealed that concentrations of cat and dog allergens in schools were higher than in homes without pets. Similar results were obtained by other authors (Dybendal and Elsayed 1994; Perzanowski et al. 1999).

Households with pets constitute a significant site of exposure to cat and dog allergens. Similarly to other studies (Ingram et al. 1995; Raunio et al. 1998; Fahlbusch et al. 2002; Arbes et al. 2004; Jedrychowski et al. 2008; Nicholas et al. 2008; Park et al. 2014), detectable levels of Fel d 1 and Can f 1 were found in almost all homes without pet, but allergen concentrations were significantly lower than in dwellings with pet ownership. Moreover, concentrations of Fel d 1 and Can f 1 were correlated with the number of cats and dogs kept at home. Studies conducted by Waradzyńska et al. (2012) indicated differences in concentrations of cat and dog allergens in homes of rural and urban environment. It could be explained by different conditions of animal keeping. In our study, in households with cat or dog keeping outside (like in rural areas), high or moderate levels of Fel d 1 and Can f 1 were detected in 55.6 and 22.2 % of the investigated surfaces, respectively. For comparison, in homes with cat or dog residing inside, high levels of Fel d 1 and Can f 1 were found in 70.7 and 81.8 % of total taken samples, respectively. Similar differences in concentration of allergens, depending on the pet ownership and keeping conditions were observed by other authors (Parvaneh et al. 1999; Nicholas et al. 2010). Distribution of Fel d 1 and Can f 1 can indicate preferred places of pets staying within the house (Jedrychowski et al. 2008). As could be expected, the highest concentrations of cat and dog allergens we observed in dust taken from dens of pets. Literature data show that high levels of pet allergens are often found in beds, upholstered furnishing and carpets (Chew et al. 1999; Berger et al. 2005; Jedrychowski et al. 2008; Brunetto et al. 2009; Kozajda et al. 2013). In our study, the upholstered furniture was the most frequent place of high exposure to cat allergen and the floor to dog allergen. Numerous studies have indicated that carpeted floors accumulated more allergens than smooth floors (Dybendal et al. 1991; Amr et al. 2003), but we did not observe significant differences in amounts of Fel d 1 and Can f 1 in dust from both types of flooring. Although some previous published results (Munir et al. 1994; Fahlbush et al. 1999) have indicated that dust from living rooms contained higher levels of pet allergens in comparison with other living quarters, in our study obtained concentrations were similar. It may indicate that pets spent a similar amount of time in both rooms.

Airborne Fel d 1 and Can f 1 levels were found low. In accordance with several other reports (Parvaneh et al. 2000; Custis et al. 2003; Munir et al. 2003), our results demonstrated correlation between pet allergen concentrations in settled dust and air, in homes. However, determined levels of airborne Fel d 1 and Can f 1 were lower than those obtained by other authors (Bollinger et al. 1996; Custovic et al. 1999). It could be due to the fact that majority of the measurements were conducted during the time when most of the residents were not at home. Reduced household activity resulted in a reduction of reservoir dust disturbance. As it was indicated in previous studies (Almqvist et al. 1999; Permaul et al. 2012), the levels of airborne cat and dog allergens in classrooms could be higher than in homes without a pet. In the case of dog allergen, our results correspond well with those studies. Nevertheless, concentrations of airborne Fel d 1 were below the limit of detection in all investigated schools.

We realize that our study has some limitations. A major of them is the relatively small sample size. Measurements were taken only once in each of the 17 cars, 14 classrooms and 19 dwellings. Of the investigated surfaces, only single sample was collected. Another weakness of the study is the fact that we do not have information regarding pet contacts in neighborhood.

Despite these limitations, our study has numerous strengths. While most of literature data come from only one kind of environment, our measurements were performed in both schools and homes as well as in cars. Studied dwellings and cars were divided into groups, differ in the presence of pets and use the car for animal transport. Thus, we were able to compare allergen concentrations in these environments. Furthermore, in homes and classrooms both settled dust and airborne samples were collected.

In conclusion, the highest amounts of cat and dog allergens were found in households with pets. Allergen concentrations were correlated with the number of animals kept at home. High concentrations of Fel d 1 and Can f 1, above the level that might induce acute attacks of asthma, were found in homes with pet ownership and cars of pet owners. The study revealed that cars of pet owners may be reservoirs of cat and dog allergens even when animals are not transported in them. In schools, the highest concentrations of allergens were within the moderate levels associated with increased risk of sensitization. Concentrations of cat and dog allergen in schools were higher than in homes without pet. While airborne Fel d 1 and Can f 1 levels were found low, residential allergen concentrations in settled dust and air were correlated. The presented study showed that classrooms and cars of pet owners may be important sites of exposure to cat and dog allergens, though the highest concentrations of Fel d 1 and Can f 1 are found in homes of pet owners.

## References

[CR1] Almqvist C, Larsson PH, Egmar A-C, Hedrén M, Malmberg P, Wickman M (1999). School as a risk environment for children allergic to cats and a site for transfer of cat allergen to homes. Journal of Allergy and Clinical Immunology.

[CR2] Amr S, Bollinger ME, Myers M, Hamilton RG, Weiss SR, Rossman M (2003). Environmental allergens and asthma in urban elementary schools. Annals of Allergy, Asthma and Immunology.

[CR3] Arbes SJ, Cohn RD, Yin M, Muilenberg ML, Friedman W, Zeldin DC (2004). Dog allergen (Can f 1) and cat allergen (Fel d 1) in US homes: Results from the national survey of lead and allergens in housing. Journal of Allergy and Clinical Immunology.

[CR4] Asher MI, Montefort S, Björkstén B, Lai CKW, Strachan DP, Weiland SK (2006). Worldwide time trends in the prevalence of symptoms of asthma, allergic rhinoconjunctivitis, and eczema in childhood: ISAAC phases one and three repeat multicountry cross-sectional surveys. Lancet.

[CR5] Berge M, Munir AK, Dreborg S (1998). Concentration of cat (Fel d 1), dog (Can f 1) and mite (Der f 1 and Der p 1) allergens in the clothing and school environment of Swedish schoolchildren with and without pets at home. Pediatric Allergy and Immunology.

[CR6] Berger I, Schierl R, Ochmann U, Egger U, Scharrer E, Nowak D (2005). Concentrations of dust, allergens and endotoxin in stables, living rooms and mattresses from cattle farmers in southern Bavaria. Annals of Agricultural and Environmental Medicine.

[CR7] Bollinger ME, Peyton A, Eggleston A, Flanagan E, Wood RA (1996). Cat antigen in homes with and without cats may induce allergic symptoms. Journal of Allergy and Clinical Immunology.

[CR8] Brożek GM, Zejda JE, Kowalska M, Gębuś M, Kępa K, Igielski M (2010). Opposite trends of allergic disorders and respiratory symptoms in children over a period of large-scale ambient air pollution decline. Polish Journal of Environmental Studies.

[CR9] Brunekreef B, Von Mutius E, Wong G, Odhiambo J, Garcia-Marcos L, Foliaki S (2012). Exposure to cats and dogs, and symptoms of asthma, rhinoconjunctivitis, and eczema. Epidemiology.

[CR10] Brunetto B, Barletta B, Brescianini S, Masciulli R, Perfetti L, Moscato G (2009). Differences in the presence of allergens among several types of indoor environments. Annali dell’Istituto Superiore di Sanità.

[CR11] Brussee JE, Smit HA, van Strien RT, Corver K, Kerkhof M, Wijga AH (2005). Allergen exposure in infancy and the development of sensitization, wheeze and asthma at 4 years. Journal of Allergy and Clinical Immunology.

[CR12] Chew GL, Higgins KM, Gold DR, Muilenberg ML, Burge HA (1999). Monthly measurements of indoor allergens and the influence of housing type in a northern US city. Allergy.

[CR13] Custis NJ, Woodfolk JA, Vaughan JW, Platts-Mills TAE (2003). Quantitative measurement of airborne allergens from dust mites, dogs, and cats using an ion-charging device. Clinical and Experimental Allergy.

[CR14] Custovic A, Green R, Taggart SCO, Smith A, Pickering CAC, Chapman MD (1996). Domestic allergens in public places II: Dog (Can f 1) and cockroach (Bla g 2) allergens in dust and mite, cat, dog and cockroach allergens in the air in public buildings. Clinical and Experimental Allergy.

[CR15] Custovic A, Simpson A, Bardin PG, Le Souёf P (2010). Allergy is an important factor in asthma exacerbation: A Pro/Con debate. Respirology.

[CR16] Custovic A, Simpson B, Simpson A, Hallam C, Craven M, Woodcock A (1999). Relationship between mite, cat, and dog allergens in reservoir dust and ambient air. Allergy.

[CR17] Cyprowski M, Buczyńska A, Szadkowska-Stańczyk I (2013). Indoor allergens in settled dust from kindergartens in city of Łódź, Poland. International Journal of Occupational Medicine and Environmental Health.

[CR18] De Marco R, Cappa V, Accordini S, Rava M, Antonicelli L, Bortolami O (2012). Trends in the prevalence of asthma and allergic rhinitis in Italy between 1991 and 2010. European Respiratory Journal.

[CR19] Dybendal T, Elsayed S (1994). Dust from carpeted and smooth floors. VI. Allergens in homes compared with those in schools in Norway. Allergy.

[CR20] Dybendal T, Wedberg WC, Elsayed S (1991). Dust from carpeted and smooth floors: IV. Solid material, proteins and allergens collected in the different filter stages of vacuum cleaners after ten days of use in school. Allergy.

[CR21] Fahlbusch B, Gehring U, Richter K, Wichmann HE, Heinrich J (2002). Predictors of cat allergen (Fel d 1) in house dust of German homes with/without cats. Journal of Investigational Allergology and Clinical Immunology.

[CR22] Fahlbush B, Heinrich J, Groß I, Jäger L, Richter K, Wichmann H-E (1999). Allergens in house-dust samples in Germany: Results of an East-West German comparison. Allergy.

[CR23] Fsadni P, Montefort S (2013). School indoor air quality and allergen exposure. Malta Medical Journal.

[CR24] Gent JF, Belanger K, Triche EW, Bracken MB, Beckett WS, Leaderer BP (2009). Association of pediatric asthma severity with exposure to common household dust allergens. European Respiratory Journal.

[CR25] Heinrich J (2011). Influence of indoor factors in dwellings on the development of childhood asthma. International Journal of Hygiene and Environmental Health.

[CR26] Ingram JM, Sporik R, Rose G, Honsinger R, Chapman MD, Platts-Mills TAE (1995). Quantitative assessment of exposure to dog (Can f 1) and cat (Fel d 1) allergens: Relation to sensitization and asthma among children living in Los Alamos, New Mexico. Journal of Allergy and Clinical Immunology.

[CR27] Instanes C, Hetland G, Berntsen S, Lovik M, Nafstad P (2005). Allergens and endotoxin in settled dust from day-care centers and schools in Oslo, Norway. Indoor Air.

[CR28] Janson C, Anto J, Burney P, Chinn S, de Marco R, Heinrich J (2001). The European community respiratory health survey: What are the main results so far?. European Respiratory Journal.

[CR29] Jedrychowski W, Perera FP, Maugeri U, Zembala M, Hajto B, Flak E (2008). Validity of the interview on pets kept at home for predicting the actual domestic exposure to their specific allergens. Krakow inner city area study. Central European Journal of Medicine.

[CR30] Johnson CC, Ownby DR, Zoratti EM, Alford SH, Williams LK, Joseph CLM (2002). Environmental epidemiology of pediatric asthma and allergy. Epidemiologic Revives.

[CR31] Justino CM, Segundo GR, Pereira FL, Silva DAO, Sopelete MC, Sung SS (2005). Mite and pet allergen exposure in Brazilian private cars. Annals of Allergy, Asthma and Immunology.

[CR32] Karlsson AS, Renström A (2005). Human hair is a potential source of cat allergen contamination of ambient air. Allergy.

[CR33] Karlsson AS, Renström A, Hedrén M, Larsson K (2004). Allergen avoidance does not alter airborne cat allergen levels in classrooms. Allergy.

[CR34] Kasznia-Kocot J, Kowalska M, Górny RL, Niesler A, Wypych-Ślusarska A (2010). Environmental risk factors for respiratory symptoms and childhood asthma. Annals of Agricultural and Environmental Medicine.

[CR35] Kozajda A, Bródka K, Sowiak M, Sobala W, Polańska K, Jurewicz J (2013). Children’s residential exposure to selected allergens and microbial indicators: Endotoxins and (1 → 3)-ß-d-glucans. International Journal of Occupational Medicine and Environmental Health.

[CR36] Leaderer BP, Belanger K, Triche E, Holford T, Gold DR, Kim Y, Jankun T, Ren P, McSharry JE, Platts-Mills TA, Chapman MD, Bracken MB (2002). Dust mite, cockroach, cat, and dog allergen concentrations in homes of asthmatic children in the northeastern United States: Impact of socioeconomic factors and population density. Environmental Health Perspectives.

[CR37] Liebhart J, Malolepszy J, Wojtyniak B, Pisiewicz K, Plusa T, Gładysz U (2007). Prevalence and risk factors for asthma in Poland: Results from the PMSEAD study. Journal of Investigational Allergology and Clinical Immunology.

[CR38] Majkowska-Wojciechowska B, Pełka J, Korzon L, Kozłowska A, Kaczała M, Jarzębska M (2007). Prevalence of allergy, patterns of allergic sensitization and allergy risk factors in rural land Urban children. Allergy.

[CR39] Maziak W, Behrens T, Brasky TM, Duhme H, Rzehak P, Weiland SK (2003). Are asthma and allergies in children and adolescents increasing? Results from ISAAC phase I and phase III surveys in Mϋnster, Germany. Allergy.

[CR40] Munir AK, Björkstén B, Einarsson R, Schou C, Ekstrand-Tobin A, Warner A (1994). Cat (Fel d 1), dog (Can f 1), and cockroach allergens in homes of asthmatic children from three climatic zones in Sweden. Allergy.

[CR41] Munir AKM, Einarsson R, Dreborg S (2003). Variability of airborne cat allergen, Fel d 1, in public place. Indoor Air.

[CR42] Murray CS, Woodcock A, Custovic A (2001). The role of indoor allergen exposure in the development of sensitization and asthma. Current opinion in Allergy and Clinical Immunology.

[CR43] Neal JS, Arlian LG, Morgan MS (2002). Relationship among house-dust mites, Der 1, Fel d 1, and Can f 1 on clothing and automobile seats with respect to densities in houses. Annals of Allergy, Asthma and Immunology.

[CR44] Nicholas C, Wegienka G, Havstad S, Ownby D, Johnson CC (2008). Influence of cat characteristics on Fel d 1 levels in the home. Annals of Allergy, Asthma and Immunology.

[CR45] Nicholas C, Wegienka G, Havstad S, Zoratti E, Ownby D, Johnson CC (2010). Dog characteristic and dog allergen levels in the home. Annals of Allergy, Asthma and Immunology.

[CR46] Nicolaou N, Siddique N, Custovic N (2005). Allergic disease in urban and rural populations: Increasing prevalence with urbanization. Allergy.

[CR47] Park HJ, Lee J-H, Park KH, Ann HW, Jin M-N, Choi S-Y (2014). A nationwide survey of inhalant allergens sensitization and levels of indoor major allergens in Korea. Allergy, Asthma and Immunology Research.

[CR48] Parvaneh S, Ahlf E, Elfman LHM, van Hage-Hamsten M (2000). A new method for collecting airborne allergens. Allergy.

[CR49] Parvaneh S, Kronqvist M, Johansson E, van Hage-Hamsten M (1999). Exposure to an abundance of cat (Fel d 1) and dog (Can f 1) allergens in Swedish farming households. Allergy.

[CR50] Permaul P, Hoffman E, Fu C, Sheehan W, Baxi S, Gaffin J, Lane J (2012). Allergens in urban schools and homes of children with asthma. Pediatric Allergy and Immunology.

[CR51] Perzanowski MS, Rönmark E, Nold B, Lundbäck B, Platts-Mills TA (1999). Relevance of allergens from cats and dogs to asthma in the northernmost province of Sweden: School as major site of exposure. Journal of Allergy and Clinical Immunology.

[CR52] Platts-Mills TA, Woodfolk JA (2011). Allergens and their role in the allergic immune response. Immunological Rewiews.

[CR53] Qu F, Weschler LB, Sundell J, Zhang Y (2013). Increasing prevalence of asthma and allergy in Beijing pre-school children: Is exclusive breastfeeding for more than 6 months protective. Chinese Science Bulletin.

[CR54] Raunio P, Pasanen A-L, Reiman M, Virtanen T (1998). Cat, dog, and house-dust-mite allergen levels of house dust in Finnish apartments. Allergy.

[CR55] Salo PM, Arbes SJ, Crockett PW, Thorne PS, Cohn RD, Zeldin DC (2008). Exposure to multiple indoor allergens in US homes and its relationship to asthma. Journal of Allergy and Clinical Immunology.

[CR56] Salo PM, Sever ML, Zeldin DC (2009). Indoor allergens in school and day care environments. Journal of Allergy and Clinical Immunology.

[CR57] Samoliński B, Sybilski AJ, Raciborski F, Tomaszewska A, Samel-Kowalik P, Walkiewicz A (2009). Prevalence of asthma in children, adolescents and young adults in Poland—results of the ECAP study. Allergy Asthma Immunology.

[CR58] Taketomi EA, Justino CM, Pereira FL, Segundo GR, Sopelete SJ, Sung SJ (2006). Taxis but not private cars are mite allergen reservoirs in Brazil. Journal of Investigational Allergology and Clinical Immunology.

[CR59] Tranter DC (2005). Indoor allergens in settled school dust: A review of findings and significant factors. Clinical and Experimental Allergy.

[CR60] Waradzyńska A, Majkowska-Wojciechowska B, Pełka J, Korzon L, Kaczała M, Jarzębska M (2012). Association of house dust allergen concentrations with residential conditions in city and in rural houses. World Allergy Organization Journal.

[CR61] Wciórka, B. (2003). Report: Looking after pets during summer holiday. *Public Opinion Research Center.* http://www.cbos.pl/SPISKOM.POL/2003/K_13_03.PDF.

[CR62] Zahradnik E, Raulf M (2014). Animal allergens and their presence in the environment. Frontiers in Immunology.

